# Impaired damping of cerebral blood flow velocity pulsatility is associated with the number of perivascular spaces as measured with 7T MRI

**DOI:** 10.1177/0271678X231153374

**Published:** 2023-01-26

**Authors:** Marieke van den Kerkhof, Merel M van der Thiel, Robert J van Oostenbrugge, Alida A Postma, Abraham A Kroon, Walter H Backes, Jacobus FA Jansen

**Affiliations:** 1 Department of Radiology & Nuclear Medicine, Maastricht University Medical Center, Maastricht, The Netherlands; 2 School for Mental Health and Neuroscience, Maastricht University, Maastricht, The Netherlands; 3 Department of Psychiatry & Neuropsychology, Maastricht University, Maastricht, The Netherlands; 4 Department of Neurology, Maastricht University Medical Center, Maastricht, The Netherlands; 5 Cardiovascular Research Institute Maastricht (CARIM), Maastricht University, Maastricht, The Netherlands; 6 Department of Internal Medicine, Maastricht University Medical Center, Maastricht, The Netherlands; 7 Department of Electrical Engineering, Eindhoven University of Technology, Eindhoven, The Netherlands

**Keywords:** 7T MRI, blood flow velocity pulsatility, damping factor, lenticulostriate arteries, perivascular spaces

## Abstract

Arterial walls stiffen with age, cardiovascular risk factors, and various vascular diseases, which may lead to less damping of the arterial blood flow pulse, subsequent microvascular damage, and enlarged perivascular spaces (PVS). However, the exact interplay between these processes is unclear. This study aimed to investigate the relation between blood flow velocity pulsatility in the small lenticulostriate arteries and their supplying middle cerebral artery and the respective damping factor (DF), with the number of MRI-visible PVS in elderly subjects. Blood flow velocity waveforms were obtained in 45 subjects (median age [range]: 64 [48–81] years, 47% male) using 7T MRI. PVS were scored in the basal ganglia (BG) and centrum semiovale (CSO). Spearman correlation analyses were used to determine associations of the blood flow pulsatility and the DF, with PVS score, adjusted for age and sex. We found a significant association between a lower DF and a higher number of PVS in the BG (*r_s_* = −0.352, *P* = 0.021), but not in the CSO. This finding supports the supposed pathophysiological mechanism in which excessive kinetic energy deposition leads to damage of small perforating arteries and contributes to the enlargement of PVS at the level of the BG, but possible other pathways might also be of influence.

## Introduction

Arterial stiffening is a critical alteration that occurs during ageing and exposure to cardiovascular risk factors, and accelerates in various (cerebro)vascular diseases.^1–4^ A consequence of stiffening of the large arteries is a decreased damping of the energetic blood flow pulse. As a result, the transmission of excessive pulsatile energy into the cerebral microcirculation is enhanced and could subsequently lead to damage of the small perforating blood vessels and its surrounding tissue.^5^

The severity of vessel stiffening can be studied by measuring blood flow velocity pulsatility, which portrays a combination of the local hemodynamics and the compliance of the vessel wall. The velocity pulsatility is commonly quantified by the pulsatility index (PI) derived from velocity measurements obtained with Transcranial Doppler (TCD) or with clinically standard 1.5 or 3T phase contrast MRI.^6–9^ However, these methods are restricted to the assessment of the largest cerebral arteries, e.g., the middle cerebral artery (MCA) with an approximate diameter of 3 mm.

Ultra-high field MRI at 7T enables to achieve a high spatial resolution and thereby allows visualization and measurement of the blood flow velocity waveform in smaller cerebral vessels, such as the lenticulostriate arteries (LSAs; approximate diameter 1.5 mm).^10–12^ The LSAs branch from the MCA and perforate the deep brain tissue. Hence, with increased blood flow velocity pulsatility, the (subcortical) brain tissue surrounding the LSAs becomes more susceptible to damage.

The role of the increased pulsatility in the development of microvascular damage has already been described in previous studies.^13,14^ One example of microvascular damage is the enlargement of perivascular spaces (PVS), one of the markers for cerebral small vessel disease (cSVD).^15,16^ PVS are fluid-filled tubular structures surrounding intracerebral blood vessels and are mainly identified with MRI in the basal ganglia (BG) and centrum semiovale (CSO) as hyperintensities on T_2_-weighted images.^12^ The susceptibility of PVS to vascular alterations has been known for many years, yet the anatomy and pathophysiology of PVS is complicated and still unclear.^13^

Previous studies sought to gain more understanding about the pathophysiology of PVS, by investigating the relation between vascular pulsatility measures and the enlargement of PVS.^9,17,18^ For instance, Nam et al., (2020) identified a higher blood flow velocity pulsatility of a large intracranial vessel – the MCA – as quantified with TCD, in subject groups with a higher PVS score, as assessed with MRI.^9^ Other pulsatility studies utilized the potential of high spatial resolution of 7 Tesla MRI to zoom into the blood flow velocity profiles of small perforating cerebral vessels, such as the LSAs.^11,12,19^ However, no previous MRI studies have specifically examined the association between pulsatility characteristics of the small LSAs and the spatially surrounding PVS.

The current study aims to investigate the interplay between the blood flow velocity pulsatility and the number of MRI-visible PVS. Firstly, we investigate the association between velocity pulsatility measures of the MCA and LSA with the number of MRI-visible PVS obtained in two spatially distinct regions, the BG and CSO. Secondly, the association of the ratio of the velocity pulsatility of the MCA and the LSA, as represented by the damping factor (DF), with the number of MRI-visible PVS will be investigated. The DF constitutes the damping of kinetic energy down the arterial tree and a lower DF is therefore a proxy of the increased energy transmission due to vessel wall stiffening. This measure reflects the damping properties of the whole trajectory between a supplying blood vessel and its branching vessel.^1,20^ Lastly, the possible influence of important cardiovascular risk factors on the relation were considered.

## Methods

### Study population

In this study, 54 subjects were initially included. Subjects were recruited between November 2019 and June 2021 by means of advertisements on the website of Alzheimer Nederland and at the Maastricht University Medical Center, and via a recruitment website (hersenonderzoek.nl).

Exclusion criteria were a history of cerebrovascular diseases, transient ischemic attack less than 3 months ago, diagnosed dementia, diabetes mellitus, BMI >32 kg/m^2^, and the inability to undergo 7T MRI. The study was approved by the local Medical Ethical Committee of Maastricht University Medical Center, followed the ethical guidelines of the Dutch Medical Research Involving Human Subjects Act (WMO) and was in line with the Helsinki Declaration of Human Rights. This study was registered at trialregister.nl (ID: NL7537, date of registration: 2019-02-20; ID: NL8798, date of registration: 2020-07-24). Written informed consent was provided by all subjects before study participation.

### Cardiovascular risk factors

The following baseline characteristics were recorded: age, sex, hypertension, pulse pressure, Body Mass Index (BMI), history of smoking, and alcohol intake. Hypertension status (yes/no) was defined when the average of the last five measurements of the 30-minute blood pressure measurement was ≥135 mmHg systolic or ≥85 mmHg diastolic, or both, or when patients were taking antihypertensive medication. Pulse pressure was defined as the systolic blood pressure minus diastolic blood pressure. BMI was based on self-reported weight and height. History of smoking (yes/no) and alcohol intake were determined by self-reported answer, the latter being defined by the average number of consumed units of alcohol per week. History of smoking was used as a covariate, rather than the current smoking status, as none of the subject were current smokers.

### Image acquisition

Images were acquired on a 7 T MRI system (Magnetom, Siemens Healthineers, Erlangen, Germany) using a 32-channel phased-array head coil. Dielectric pads were placed on both sides of the subject’s head, in proximity to the temporal lobes, for improvement of ${\text{B}}_{1}^{+}$ field homogeneity across the brain.

**Figure 1.**
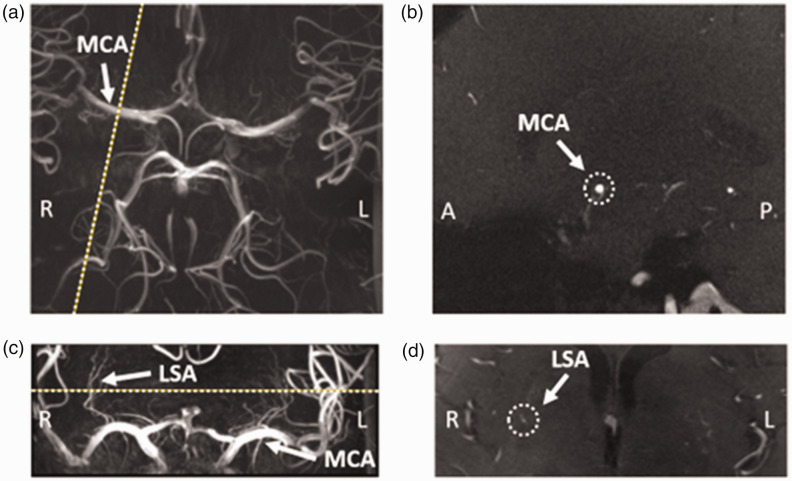
Example images from a representative subject (60 years old, female), showing the planning of the phase-contrast MRI (PC-MRI) slice on the middle cerebral artery (MCA) and the lenticulostriate artery (LSA). (a) Time-Of-Flight image at the level of the Circle of Willis, showing the MCA (arrow). The planning of the PC-MRI slice on the right MCA is indicated by the dotted line. (b) PC-MRI image showing the planned slice indicated in a, with the MCA indicated by the dotted circle. (c) Coronal Maximum Intensity Projection image showing the MCAs (arrow) and LSAs (arrow). Planning of the PC-MRI slice is indicated by the dotted line. (d) PC-MRI image showing the planned slice indicated in c, highlighting the chosen LSA on the right of the brain (inside the dotted circle).

First, a time-of-flight angiogram was acquired for the 3D depiction of the branching and trajectories of the MCAs and LSAs (detailed scan parameters are provided in Table 1). Subsequently, maximum intensity projections were calculated for the geometrical planning of the slices that would acquire the blood flow velocities (Figure 1).

**Table 1.** Acquisition parameters of the sequences.

*Acquisition parameters*	Angiogram	LSA	MCA	T_2_-weighted	T_1_-weighted
*Sequence*	TOF	PC-MRI	PC-MRI	Turbo spin-echo	MP2RAGE
*TR [ms]*	15.0	50–70	44.5	4000	5000
*TE [ms]*	5.1	4–5	4.1	283	2.47
*VENC [cm/s]*	–	30	100	–	–
*Field-of-view [mm^2^]*	135 × 180	180 × 180	180 × 180	192 × 192	224 × 224
*Voxel size [mm^3^]*	0.31 × 0.31 × 0.31	0.31 × 0.31 × 2.6	0.28 × 0.28 × 2.0	0.6 × 0.6 × 2.0	0.7 × 0.7 × 0.7
*Bandwidth [Hz/pixel]*	78	181–280	280–434	372	250
*Flip angle [◦]*	18	26	27	120	5/3
*Acquisition time [min:s]*	4:49	4:00	3:30	3:34	8:00

Second, 2D PC MRI was applied for the measurement of the blood flow velocity waveforms in the LSAs (Table 1). To achieve the highest signal-to-noise ratio possible, while maintaining a minimum of 16 cardiac phases, the bandwidth, TR, and TE were set to the lowest feasible values. The system’s acquisition window per cardiac cycle was approximately 920 ms, which means that for the MCA the temporal resolution was around 45 ms and for the LSAs 50–70 ms. The velocity encoding (VENC) was set to 30 cm/s. No imaging acceleration factors were used, as this would lower the signal-to-noise ratio which would especially be disadvantageous for LSA measurements.

Third, a similar 2D PC MRI sequence was applied (Table 1), to measure blood flow velocity waveforms in the ipsilateral, feeding MCA of the largest identified LSA, for which approximately 20 cardiac phases were obtained. The VENC was set to 100 cm/s, however, when aliasing was noticed during the scan as a result of exceeding the VENC, the scan was repeated with a VENC of 120 cm/s when there was sufficient scanning time. The slices of both PC MRI sequences were planned perpendicular to the vessel of interest (Figure 1) and the scans were prospectively gated with a trigger provided by a pulse oximeter placed on the subject’s finger.

Fourth, for visualization of the MRI-visible PVS, a whole-brain T_2_-weighted turbo spin echo was applied (Table 1).

Fifth, to obtain the brain size per subject, a T_1_-weighted Magnetization-Prepared 2 Rapid Acquisition Gradient Echoes (MP2RAGE) was performed (Table 1).

### Image analysis

#### Vessel selection

The largest LSA and its feeding MCA were identified for each individual subject. The corresponding blood flow velocity waveforms were obtained by transforming the phase images into velocities for each voxel. When the velocity of the vessel exceeded the VENC, automated aliasing correction was applied.^21^ Furthermore, both the phase and magnitude images were corrected for background noise by calculating the mean noise level in a region of the static brain tissue near the artery of interest. Only voxels with intensity values above this noise threshold were used for further analysis. Subsequently, the vessel was automatically segmented using the magnitude images and the corresponding voxels in the phase images were selected to quantify the blood flow velocities.

#### Velocity measures

The voxel representing the peak velocity – defined as highest mean velocity over one cardiac cycle – of the MCA and the largest LSA were identified. The pixel with the highest (mean) velocity is relatively less influenced by noise compared to pixels at the border of the vessel, especially considering the pulsatile movement of vessels which causes variations in the location of pixels and area of the vessel between phases. By using the pixel containing the highest mean velocity, we ascertain that this pixel is included within the area of the vessel in each phase. Furthermore, we ensure a systematic approach and thereby reduce variability within our analyses. The time-averaged blood flow velocity was computed from the corresponding velocity waveform. Moreover, the PI was calculated for each vessel using Gosling’s equation: $\mathrm{PI}=\frac{v_{\max}-v_{\min}}{v_{\mathrm{mean}}}$ , where *v*_max_ is the maximum, *v*_min_ is the minimum, and *v*_mean_ is the mean velocity.^7^ In addition, the DF was derived as a measure of vessel compliance and was defined as the ratio of the PI of the MCA and the LSA (DF = $\frac{\mathrm{PI}\;\mathrm{in}\;\mathrm{the}\;\mathrm{MCA}}{\mathrm{PI}\;\mathrm{in}\;\mathrm{the}\;\mathrm{LSA}}$ ), which is inversely related to vessel wall stiffness.

All image analyses were performed using Matlab (2019 b, 9.2.0; Mathworks, Nattick, MA, USA).

#### PVS scoring

**Figure 2.**
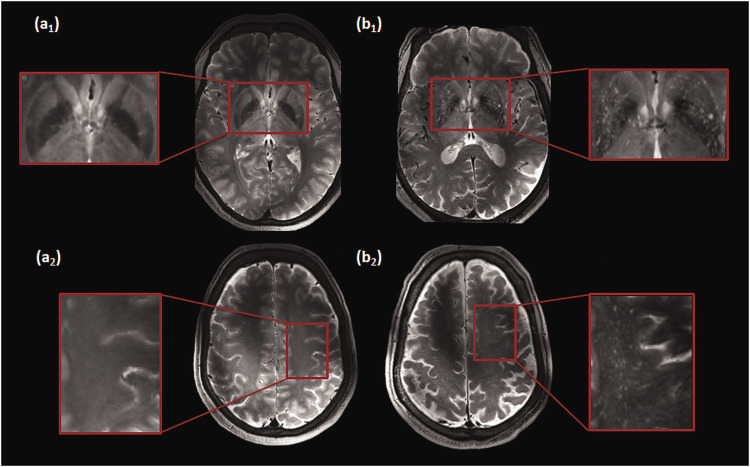
Representative T_2_-weighted images for the scoring of the perivascular spaces. (a) Example images of a subject showing a low number of MRI-visible PVS in the basal ganglia (a_1_) and the centrum semiovale (a_2_) (magnified areas are shown in red boxes). (b) Example images of a subject showing a large number of MRI-visible PVS in the basal ganglia (b_1_) and the centrum semiovale (b_2_) (magnified areas are shown in red boxes).

The MRI-visible PVS for each subject were rated on the slice with the highest number of PVS, in both the BG and CSO, in one hemisphere. Initially, the score was stratified in 4 groups: 0 ≤ 10, 1 = 11–25, 2 = 26–40, 3 ≥ 41 PVS, based on a combination of previously published rating scales.^15,22^ Two examples of the high-resolution images with high and low MRI-visible PVS scores are displayed in Figure 2. Consensus scoring was performed by three raters; an experienced neuroradiologist (A.A.P.: >20 years of experience) and two trained neuroscientists (M.v.d.T.: 2 years of rating experience, M.v.d.K.: 1 year of rating experience). The rating resulted in a PVS score of 3 in the BG for only a single subject, and two subjects received a PVS score of 0 in the CSO. Hence, in further analyses these subjects were included in the group of the PVS scores of 2 in the BG, and 1 in the CSO, respectively, to increase the power.

#### Brain size

The T_1_-weighted images were given as input for automated brain tissue segmentation (Freesurfer, version 6.0.5).^23^ When required after visual inspection, the output was manually corrected. The brain size was retrieved from the gray and the white matter volumes.

### Statistics

Partial Spearman’s rank-order correlation coefficients were computed between the PI of the MCA and LSA, DF, and the PVS scores (IBM SPSS statistics version 25). Based on previous literature which has shown an influence of age on blood flow velocity pulsatility^1,12^ and on the number of MRI-visible PVS,^24–27^ and various studies which have shown the effect of sex on the number of MRI-visible PVS,^25–27^ the associations were adjusted for age and sex. To exclude a potential confounding effect of brain size on the relation between PVS enlargement and pulsatility measures, this analysis was repeated while additionally correcting for brain size.

Moreover, to isolate the possible influence of potential risk factors on the relation between the pulsatility measures and the PVS score (i.e., hypertension status, pulse pressure, BMI, history of smoking, alcohol intake), additional partial Spearman’s rho correlations adjusted for age and sex and each individual risk factor were assessed. A threshold level of alpha = 0.05 was used to determine statistically significant effects.

## Results

**Figure 3.**
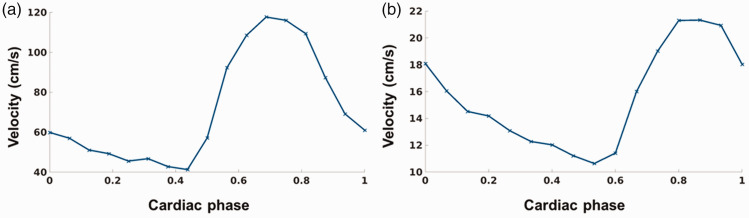
Representative blood flow velocity profiles obtained (a) in the middle cerebral artery (54 years, female) and (b) in a lenticulostriate artery (56 years, female).

Nine subjects were excluded for further analyses due to motion artefacts or incorrect slice planning for either the MCA, the LSA, or both, leading to the inclusion of 45 subjects for data analysis. We analysed these 45 participants with a median age of 65 years, of whom 47% were males and 47% had hypertension. The demographics are summarized in Table 2. The descriptive statistics of the velocity waveform measures, including the PI of the MCA and LSA, the DF, and the PVS scores are listed in Table 3. Figure 3 shows an example of the blood flow velocity profile acquired in the MCA and in the LSA.

**Table 2.** Demographical data of the included study participants.

Participant characteristics (n = 45)	
Age in years (Median [Q1–Q3])	65 [48–81]
Male, *n (%)*	21 (47)
Hypertension, *n (%)*	24 (47)
Smoking, *n (%)*	0 (0)
History of smoking*, n (%)*	19 (42)
Alcohol in units per week (Median [Q1–Q3])	3.5 [.75–7.25]
Body mass index in kg/m^2^ (Mean (SD))	25.5 (3.2)

**Table 3.** Descriptive statistics of the study parameters. Median [Q1–Q3] is reported unless stated otherwise.

PC-MRI measures (n = 45)			
LSA			
*v*_min_ [cm/s]	5.9 [4.4–7.2]		
*v*_max_ [cm/s]	12.7 [11.1–15.5]		
*v*_mean_ [cm/s]	8.4 [7.1–10.6]		
PI [–]	.85 [.73–.96]		
MCA			
*v*_min_ [cm/s]	27.6 [22.9–31.7]		
*v*_max_ [cm/s]	64.3 [56.7–78.4]		
*v*_mean_ [cm/s]	41.9 [37.9–49.9]		
PI [*–*]	.87 [.72–1.03]		
MCA to LSA			
DF [*–*]	1.03 [.90–1.20]		
PVS score (n = 45)			
PVS basal ganglia score	**0**	**≤10**	13 (28.9)
*Frequency (%)*	**1**	**11–25**	26 (57.8)
	**2**	**26–40**	5 (11.1)
	**3**	**≥41**	1 (2.2)
PVS centrum semiovale score	**0**	**≤10**	2 (4.4)
*Frequency (%)*	**1**	**11–25**	4 (8.9)
	**2**	**26–40**	21 (46.7)
	**3**	**≥41**	18 (40.0)

PC-MRI: phase contrast MRI; LSA: lenticulostriate artery; MCA: middle cerebral artery; *v*: velocity; PI: pulsatility index; DF: damping factor; PVS: (MRI-visible) perivascular spaces.

### Associations between pulsatility measures and PVS score

The results of the correlation analyses are displayed in Table 4.

**Table 4.** Spearman’s partial rank-order correlations adjusted for age and sex between MRI-visible PVS score of the basal ganglia and centrum semiovale and vessel pulsatility measures.

Spearman’s partial correlation adjusted for age and sex				
PC-MRI measures (n = 45)	PVS scorebasal ganglia		PVS scorecentrum semiovale	
	*r_s_*	*P*	*r_s_*	*P*
LSA				
*PI*	.175	.263	−.041	.792
MCA				
*PI*	−.223	.151	.095	.545
MCA to LSA				
*DF*	−**.352**	.021*	.131	.404

PVS: (MRI-visible) perivascular spaces; LSA: lenticulostriate artery; MCA: middle cerebral artery; PC-MRI: phase contrast MRI; PI: pulsatility index; DF: damping factor; r_s_: Spearman’s rho.

Significant associations are depicted in bold (*P < 0.05).

The DF was found to be significantly correlated to the PVS score in the BG (*r_s_* = −0.352, *P* = 0.021), where a lower DF related to a higher PVS score, while adjusting for age and sex. This association remained significant after additional correction for brain size (*r_s_* = −.359, *P* = 0.020). No other significant correlations of the PI of the MCA or LSA with PVS scores in either the BG or the CSO were found.

### Associations considering risk factors

When additionally correcting for pulse pressure, BMI, history of smoking, or alcohol intake, the correlation adjusted for age and sex between the DF and PVS score in the BG remained significant (*r_s_* = −.338, *P =* .029; *r_s_* = −.347, *P =* .024; *r_s_* = −.354, *P =* .022; *r_s_* = −.354, *P =* .021, respectively). However, when additionally adjusting for hypertension, only a trend remained (*r_s_* = −.259, *P =* .097). To assess the relation with pulse pressure in more detail, multiple linear regression adjusted for age and sex was applied between pulse pressure and the PI of the LSA, PI of the MCA, and the DF. The pulse pressure was found to be correlated with the PI of the LSA (β = 0.475, *P* = 0.002), and yielded a trend with the DF (β = −0.296, *P* = 0.072). No correlation with the PI of the MCA was found (*P* > 0.9).

## Discussion

This study aimed to investigate the relation between pulsatility measures determined with 7T MRI of two different parts of the cerebral arterial tree, the MCA and the branching LSAs, and the number of MRI-visible PVS in the BG and CSO. A lower DF was correlated with a higher number of MRI-visible PVS in the BG, independent of age, sex, and brain size. No further significant associations were found between pulsatility measures and the MRI-visible PVS in neither the BG nor the CSO. Cardiovascular risk factors such as BMI, history of smoking and alcohol intake did not alter the relation between MRI-visible PVS in the BG and the DF, but hypertension status did influence this association.

Previous studies have identified an effect of ageing on both pulsatility measures and number of MRI-visible PVS.^12,18,24^ This intrinsic effect of age could potentially modify associations between those two features. For example, after adjustment for age, Birnefeld (2020) did no longer find associations between cSVD score and PI of the MCA.^13^ The results of our study show that an altered DF is also related to the number of MRI-visible PVS, independent of age and sex. This supports the hypothesis that a decreased damping of the blood flow velocity pulse transmits excessive energy to the brain tissue adjacent to the LSAs, which could lead to tissue damage, such as enlargement of PVS.

This study only found an association between the DF and PVS score for the BG but not for the CSO, which suggests the presence of a local effect – as opposed to a global vascular effect. This could be due to the fact that the excessive kinetic energy of the blood is already deposited at the level of the BG before it reaches the more downstream CSO. Alternatively, while the BG are solely supplied by the MCA, the CSO is also supplied by other major cerebral blood vessels. Another explanation, at the level of the small cerebral arteries, is that the LSAs supply the BG directly, while the CSO is supplied by different small cerebral blood vessels. The pulsatility measures of the LSAs could thus directly have an effect on the surrounding PVS in the BG, while the PVS in the CSO are affected by the pulsatility measures of other perforating arteries (directly surrounded by the PVS in the CSO). There could be a relation between the DF derived from the specific perforating arteries of the CSO and one of its feeding arteries, and MRI-visible PVS in the CSO. However, we did not obtain velocity scans of the feeding arteries in the CSO, therefore we were unable to answer this question in this study. Future research could focus on the relation between the PVS in the CSO and the measurement of the specific arteries perforating the CSO, which was shown feasible in previous literature,^10^ This would provide more insight on whether the identified relation between the DF and the PVS score is specific for the BG, or is also valid for arteries located in other brain regions.

Although the relationship between the DF and MRI-visible PVS has not been previously studied, other studies did show an effect of increased arterial stiffness on the number of PVS.^18,28^ For instance, Gutierrez et al. (2019) described that a higher stiffness in the intermediary arteries (e.g., the anterior carotid artery and the MCA) modifies the association between extracranial pulsatility (in the aorta and common carotid arteries) with small MRI-visible PVS and white matter hyperintensities (WMH).^18^ These findings could indicate an interplay between the pulsatility measures of the large feeding artery and pulsatility measures of vessels more downstream in the arterial tree on the enlargement of PVS. Based on the results of Gutierrez et al. (2019) and those of the current study, we argue that the DF might be a more representative measure of vessel wall characteristics than the PI itself. In agreement with this notion, no association with PVS score was found when looking solely at the PI of the MCA or the LSA.

 The PI of both the MCA and LSA, as well as the velocities measured in this study, were similar to those found in previous literature,^1,11,12,19,20^ which shows that they are representative values. The lack of associations of PI values with PVS score, could imply that the PI itself has less influence on the deleterious effects on the brain tissue, but the altered vascular compliance leading to a change in the blood flow velocity profile from the MCA to the LSA might play a more substantial role. However, it remains unclear whether the lowered DF relating to more PVS reflects solely a less compliant MCA, a less compliant LSA or a combination of both. Therefore, the results of the current study suggest that decreased pulsatility damping due to decreased vessel compliance, and thus increased vascular stiffness, results in more energy being deposited to the LSAs and the microvasculature beyond the measurement location in the LSAs. This energy causes damage and thereby results in the enlargement of PVS. Specifically, the remaining absorbed energy by the less compliant LSA is used to temporarily dilate the LSA, where it may damage the interface between the vessel wall and brain tissue, subsequently leading more and/or enlarged PVS. Speculatively, when vessels temporarily dilate, the perivascular space may become narrower, as the fluid is incompressible. This perivascular fluid would then be pushed downstream where it might further damage this interface and lead to enlargement of the PVS. Therefore, when the damping is reduced, this perivascular pulse is stronger, impacting the interface at the downstream end. Alternatively, the damaged interface between the vessel wall and the brain tissue could lead to leakage from the vasculature, further leading to enlargement of PVS.

The observed correlations between blood flow pulsatility and PVS scores were significant, but did not fully elucidate the process of the enlargement of PVS. In addition to the effects of reduced vascular compliance, other explanations of PVS enlargement are also possible. PVS are thought to play an important role in the cerebral waste clearance system,^29^ therefore PVS dilation may indicate an impaired fluid drainage or the deposition of pathological proteins. As mentioned, another explanation for the enlargement of PVS could be fluid leakage from the vasculature, caused by degeneration of pericytes and a consequent loss of vascular integrity.^30^ Further research is needed to investigate the alternative explanations of PVS enlargement, and the potential, interactive effect between these pathways and blood flow velocity pulsatility on the enlargement of PVS.

Previous studies have looked into the relation between intracranial pulsatility and cSVD markers other than MRI-visible PVS and found significant positive associations between the PI of the MCA and WMH.^6,8,9,14,31^ While the TCD study of Nam et al. (2020) did not find a clear association between the PI of the MCA and the number of MRI-visible PVS in the BG, a relation with WMH volume was observed.^9^ Similarly, a previous study on patients with cSVD and controls has also reported a higher PI of small cerebral arteries, including the perforating arteries in the BG, in cSVD patients. However, the latter study did not look at PVS specifically.^19^ These previous studies imply that specific cSVD markers can display different relations with pulsatility measures. This could potentially be due to the inflammatory nature of WMH, while PVS enlargement may be more driven by vascular influences, like altered velocity pulsatility.^32^

Various cardiovascular risk factors are known to have a substantial influence on both blood flow measures and the number of MRI-visible PVS. For example, high blood pressure is recognized to increase arterial stiffening.^33,34^ When additionally adjusting the results for hypertension, the previously significant association between the DF and the PVS score in the BG was reduced to a trend. This indicates that hypertension has a considerable effect on both the pulsatility measures and on the enlargement of PVS. In contrast, when adjusting the correlation between the velocity pulsatility and PVS for pulse pressure, the results remained significant. This would imply that disease state contributes substantially to this process, as opposed to the current pulse pressure, which is a measure of the blood pressure at scanning time. Since hypertension status represents the history of the blood pressure state as well, this suggests a more chronic pathological process, instead of acute. The correlations with the PI of the LSA, the PI of the MCA and the DF, which were obtained to examine the relation with pulse pressure in more detail, only showed a significant correlation with the PI of the LSA, and a trend for the DF. This suggests that the PI of the MCA is dominant in the damping process of the blood flow pulse. Furthermore, the pulse pressure was correlated to the PI of the LSA, but did not alter the relation between DF and PVS. This is in contrast to the hypertension disease state, which altered this relation, suggesting that the underlying contributing pathological process is of a longer time period. Considering the other cardiovascular risk factors which were taken into account in this study, the relation between the DF and the PVS score in the BG was also not altered. This might indicate a pathological relation between these two features, independent of BMI, history of smoking and alcohol intake. However, to validate this, larger variations in cardiovascular risk factors and a larger sample size are needed to investigate this adequately.

Therefore, we recommend future studies to further investigate these relations in patients with underlying vascular pathology. When studying a more diverse and larger population, potential collinearity between the different cardiovascular risk factors should also be considered. Thereby, more knowledge could be obtained about the effect of (neuro)vascular diseases and cardiovascular risk factors on the relationship between pulsatility measures, such as the DF, and the enlargement of PVS. Furthermore, although several important cardiovascular risk factors are considered in this study, this list is not exhaustive. For example, the current study excluded confounding effects of caffeine intake, by asking all subject to refrain from caffeine intake 12 hours before scanning, but did not concretely investigate the effect of caffeine on the relationship between pulsatility measures and MRI-visible PVS. Likewise, the time of day of MRI scanning was aimed to be kept consistent across the subjects. Future studies are recommended to specifically investigate the potential effect of other influential factors, such as caffeine intake, time of day and physical activity.^25^

### Study strengths and limitations

A major strength of this study is the usage of high-resolution 7T MRI imaging, which allows for the visualization of PVS in great detail. More importantly, it enables the measurement of the blood flow velocity wave forms of the relatively small LSAs, which is not possible on clinical 1.5 T or 3 T scanners. The combination of these advanced measurements in a relatively large, homogeneous study sample enables to investigate a potential pathological pathway in the enlargement of PVS.

The PVS score used in this study was based on a combination of previous rating scales^15,22^ rather than a continuous measure. Another approach for quantifying MRI-visible PVS would be to use standardized rating scales for both regions. Alternatively, it could be beneficial to use machine learning techniques to segment the MRI-visible PVS, thereby providing a quantitative measure for PVS enlargement.^35,36^ However, these techniques are limited due to a preferred isotropic, and sufficiently small voxel size, which often substantially increases the scan time.

To further investigate the relation between pulsatility measures of small vessels and the enlargement of PVS, future studies should look which effect the pulsatility measures of a specific vessel would have on the size of the PVS surrounding this specific vessel. In this manner, the effect of pulsatility on PVS enlargement could be investigated on a very local scale. However, scan protocols should be optimized to investigate PVS size *a priori,* as a very high spatial resolution is required to limit partial volume effects on these small structures, and to determine an accurate measure of PVS size. Alternatively, image processing could be further developed and performed to estimate the relative size of MRI visible PVS that surrounding specific LSAs.

## Conclusion

This study identified an association between the reduced cerebral blood flow velocity pulse damping and a higher number of MRI-visible PVS in the BG, independent of age, sex, and brain size. This relation was shown to be influenced by hypertension status. Our findings support the idea that the excessive kinetic energy deposition of the pulsatile blood flow leads to damage of small perforating arteries and contributes to the enlargement of PVS at the level of the BG. Further studies are warranted to investigate this relation in various cardiovascular diseases, its potential effects on brain function, and possible other pathways for PVS enlargement, such as fluid leakage from the vasculature.

## Data Availability

Anonymized data that support the findings of this study are available from the corresponding author, upon reasonable request from any qualified investigator.
